# Supplementary figures and images for: Steroidogenesis and androgen/estrogen signaling pathways are altered in in vitro matured testicular tissues of prepubertal mice
Source: eLife. 2023 Dec 14;12:RP85562. doi: 10.7554/eLife.85562 (PMC10721218; doi:10.7554/eLife.85562)

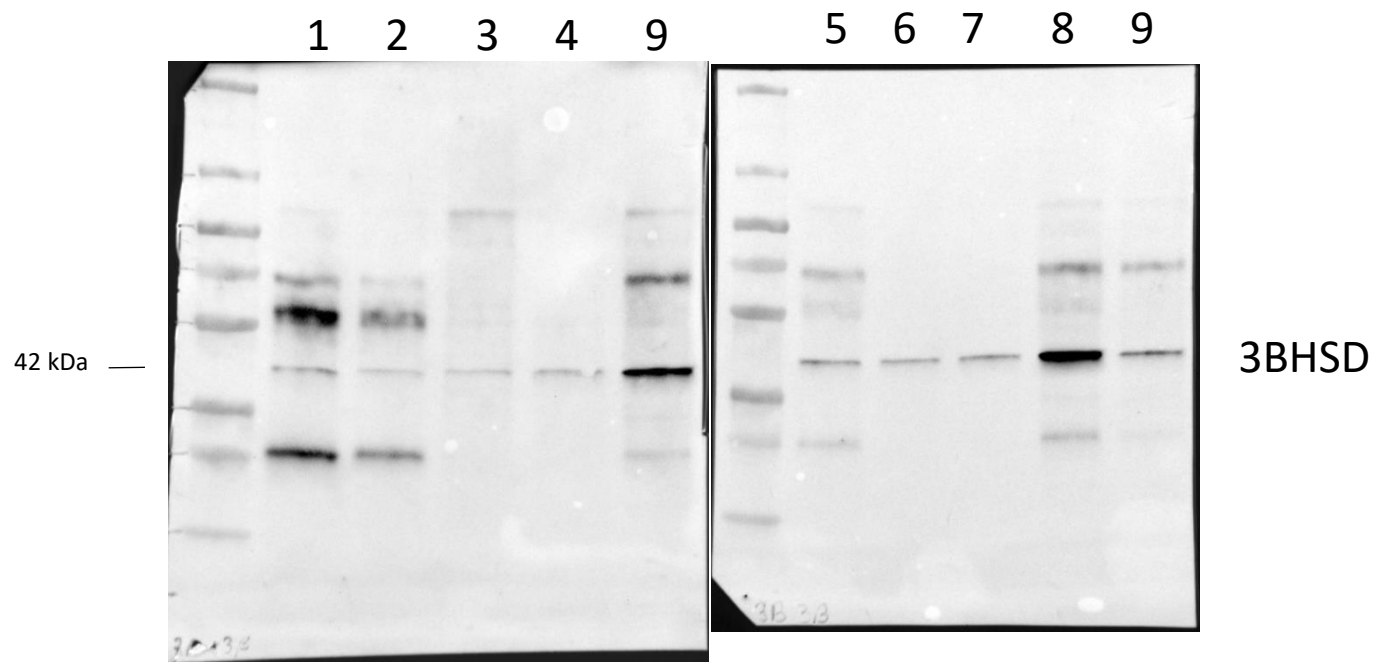

- 1 6 *dpp*
- 2 6 *dpp* CSF
- 3 D16 FT
- 4 D16 CSF
- 5 22 *dpp*
- 6 D30 FT
- 7 D30 CSF
- 8 36 *dpp*
- 9 Control

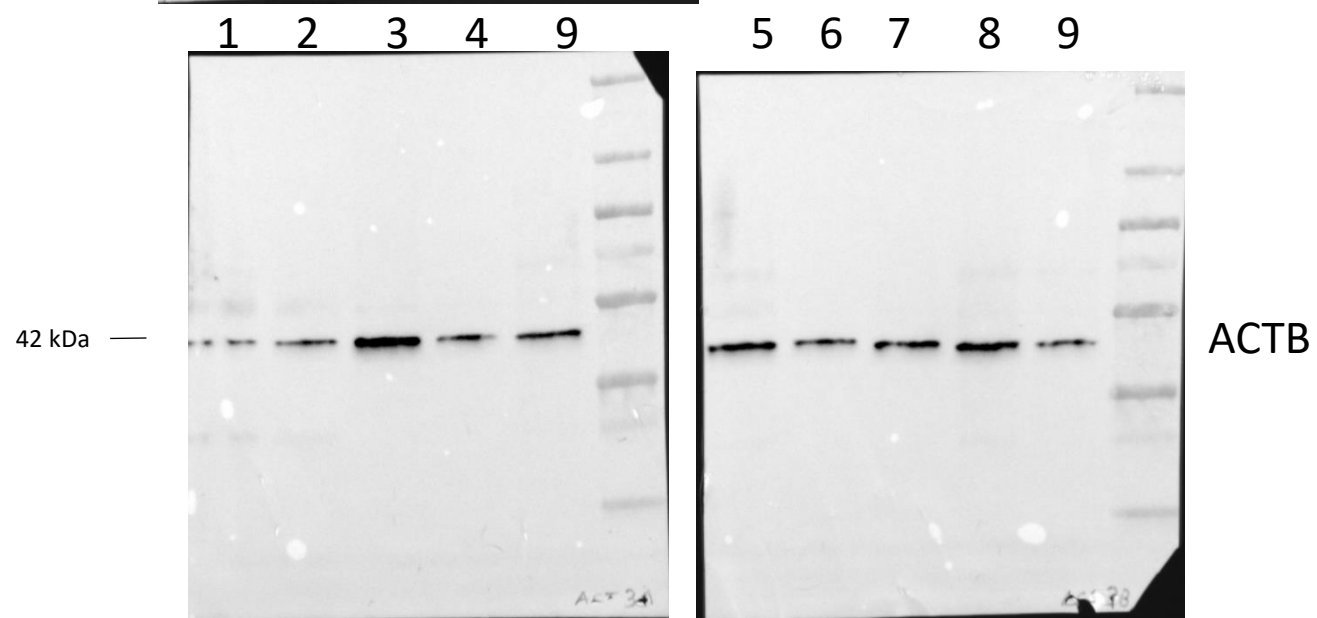

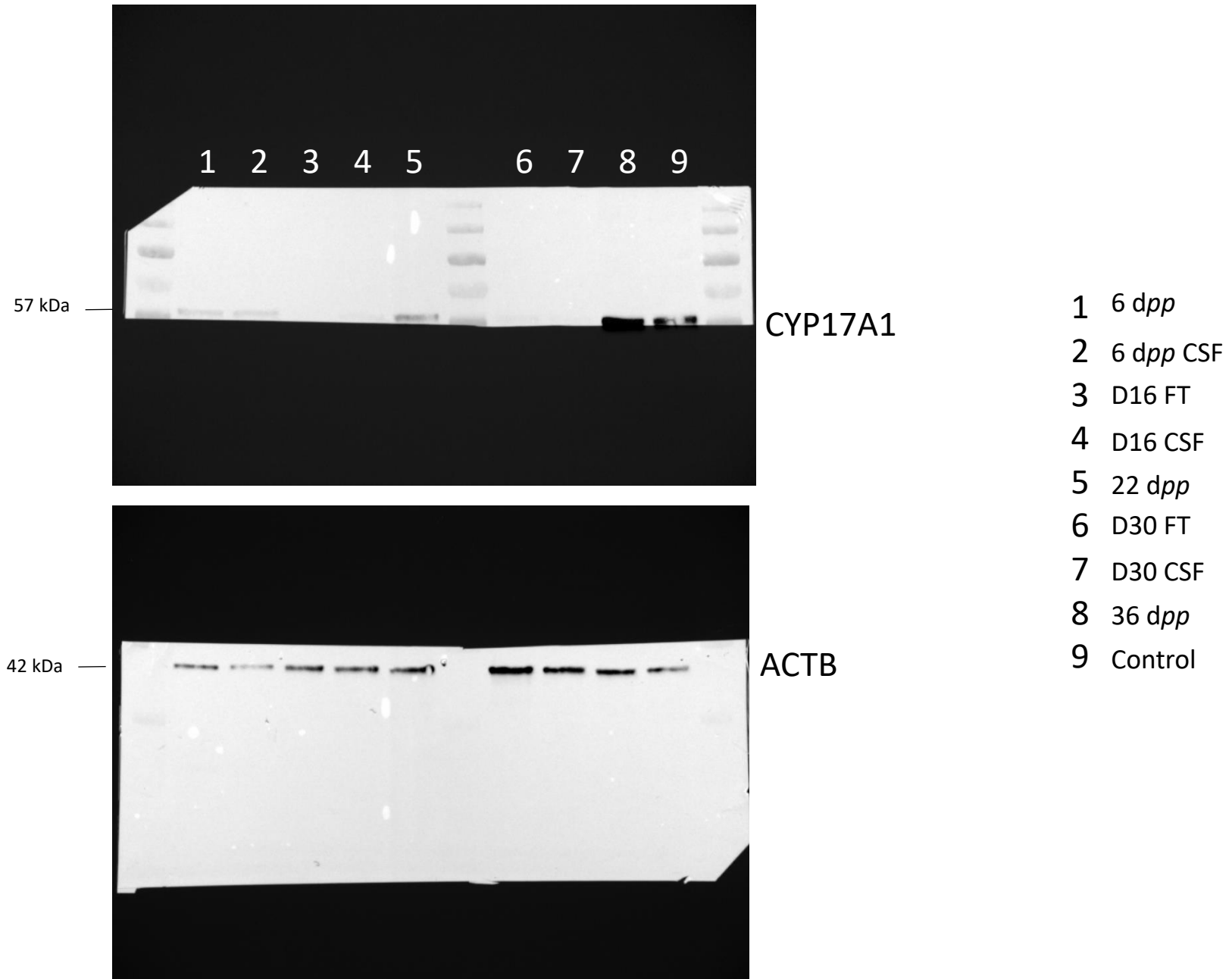

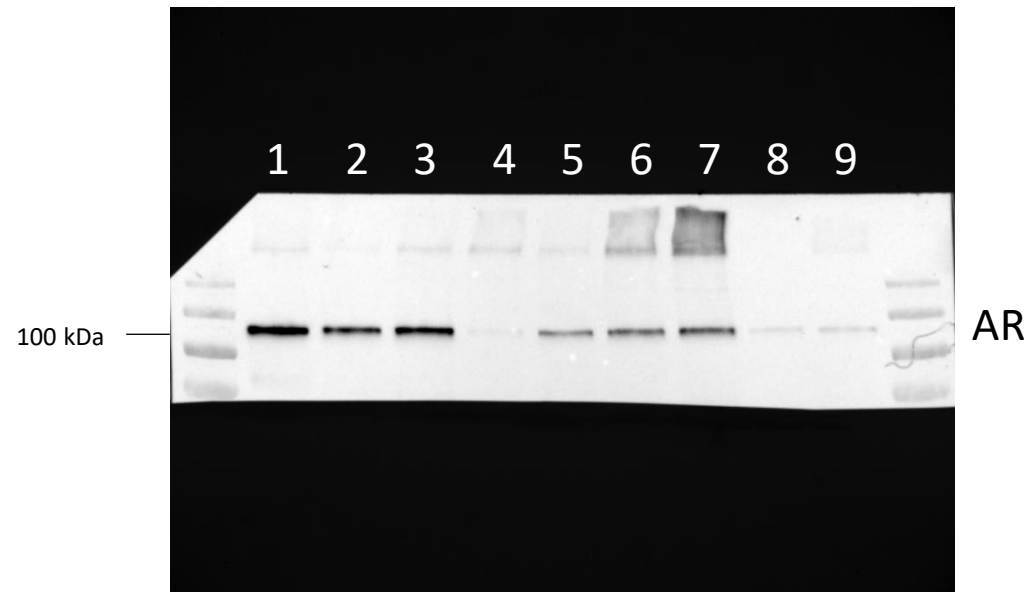

AR

- 1 6 *dpp*
- 2 6 *dpp* CSF
- 3 D16 FT
- 4 D16 CSF
- 5 22 *dpp*
- 6 D30 FT
- 7 D30 CSF
- 8 36 *dpp*
- 9 Control

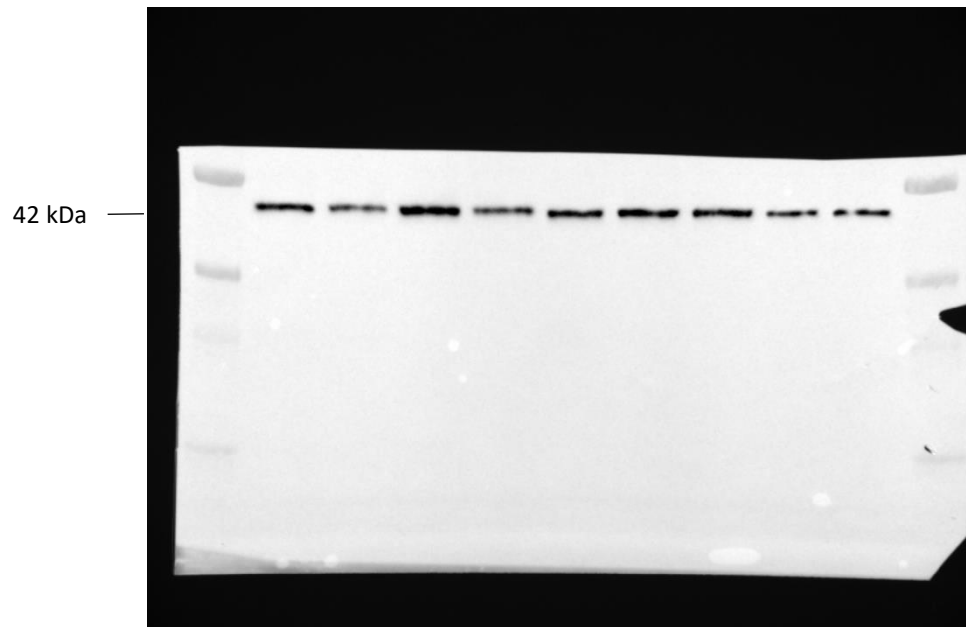

ACTB

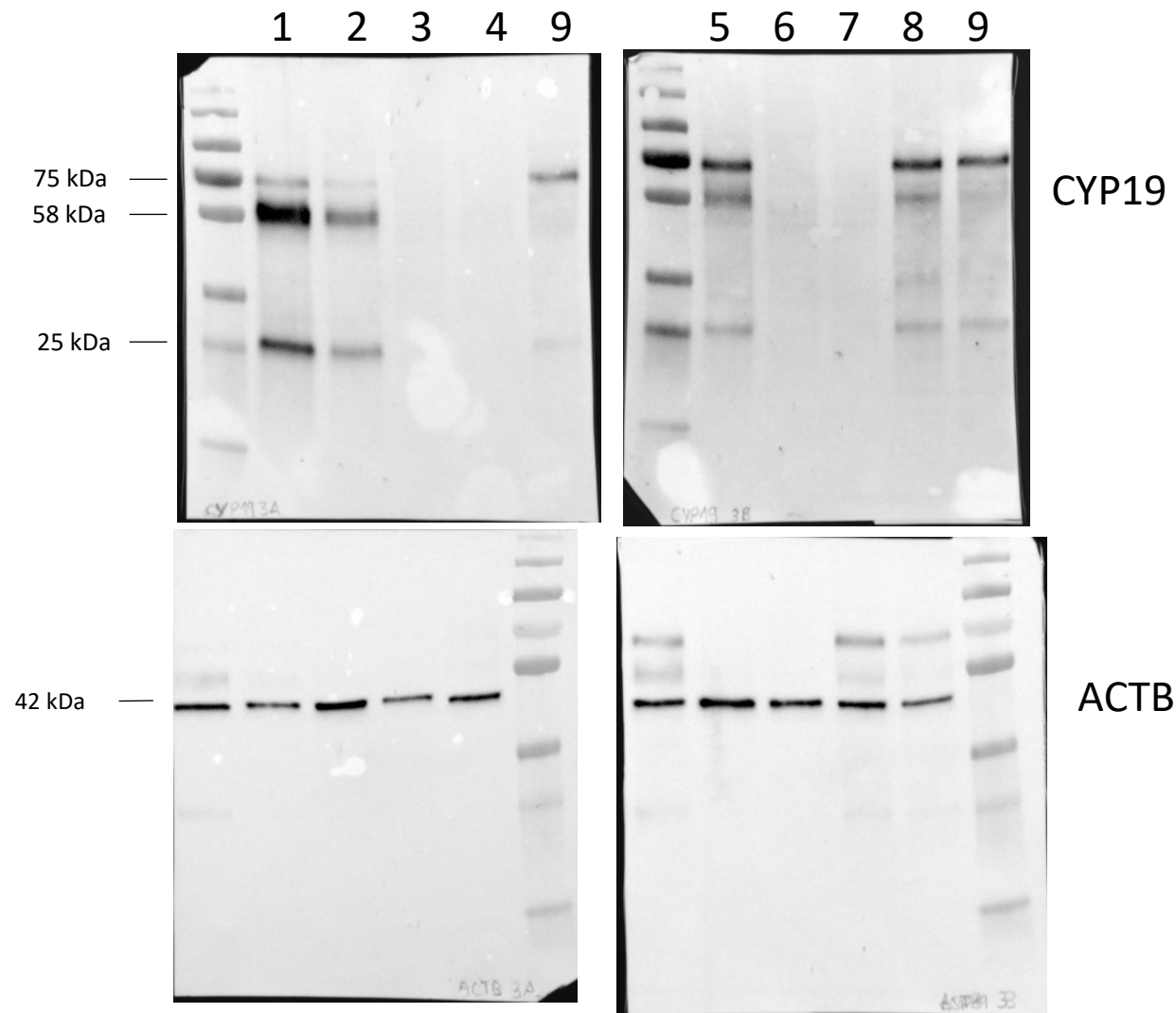

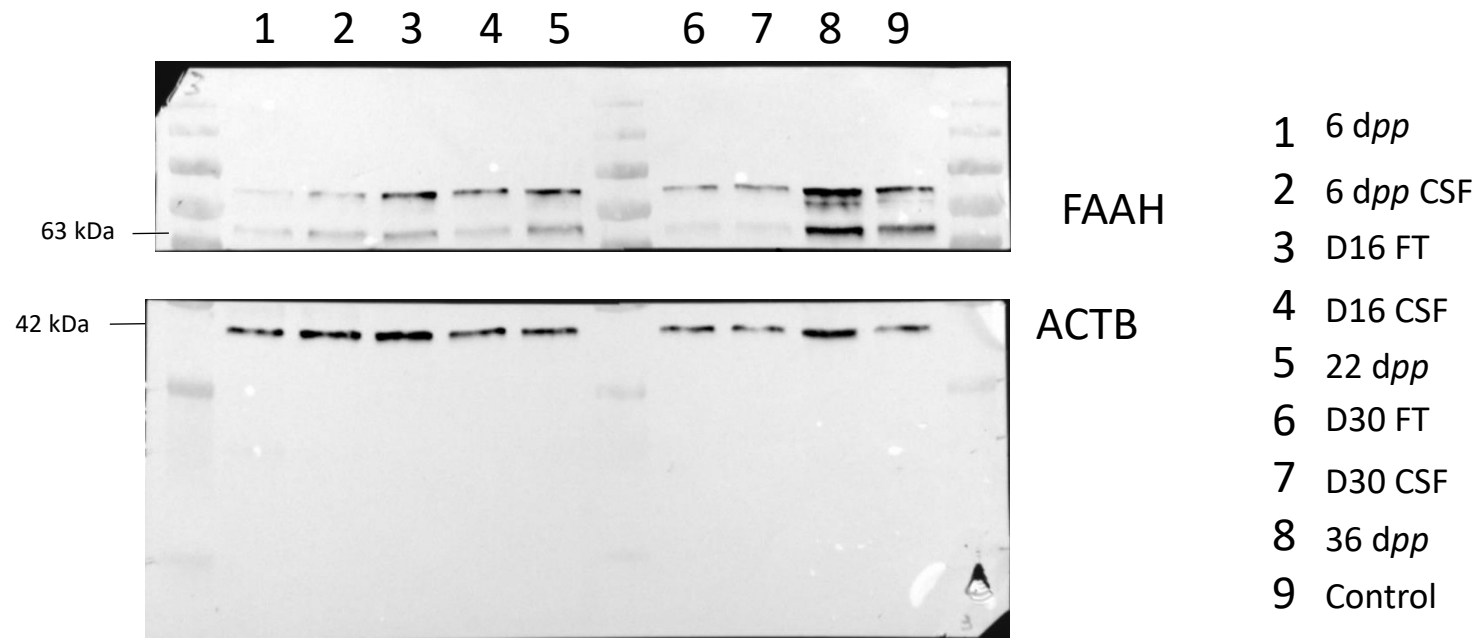

Supplement: Source data 1. [file elife-85562-data1.pdf]
